# Crime, inequality and public health: a survey of emerging trends in urban data science

**DOI:** 10.3389/fdata.2023.1124526

**Published:** 2023-05-25

**Authors:** Massimiliano Luca, Gian Maria Campedelli, Simone Centellegher, Michele Tizzoni, Bruno Lepri

**Affiliations:** ^1^Mobile and Social Computing Lab, Bruno Kessler Foundation, Trento, Italy; ^2^Faculty of Computer Science, Free University of Bolzano, Bolzano, Italy; ^3^Department of Sociology and Social Research, University of Trento, Trento, Italy

**Keywords:** cities, crime, segregation and inequalities, public health, digital data

## Abstract

Urban agglomerations are constantly and rapidly evolving ecosystems, with globalization and increasing urbanization posing new challenges in sustainable urban development well summarized in the United Nations' Sustainable Development Goals (SDGs). The advent of the digital age generated by modern alternative data sources provides new tools to tackle these challenges with spatio-temporal scales that were previously unavailable with census statistics. In this review, we present how new digital data sources are employed to provide data-driven insights to study and track (i) urban crime and public safety; (ii) socioeconomic inequalities and segregation; and (iii) public health, with a particular focus on the city scale.

## 1. Introduction

Cities occupy only 3% of the global surface but are inhabited by more than 50% of the world's population.^1^ Timely and accurate data are thus becoming fundamental for policymakers and municipalities to control cities' dynamics and respond to multiple societal challenges. In 2015, the United Nations set out 17 Sustainable Development Goals (SDGs)^2^ that summarize the new challenges we have to face to guarantee everyone a better and more sustainable future. Examples of such goals are about guaranteeing good quality of (accessible) health and wellbeing, reduction of inequalities, and design of sustainable and safe cities and communities.

It is clear that big urban agglomerations have a pivotal role in the accomplishment of such goals as many of them are fundamentally related to human movements, displacement, and interactions (Glaeser, 2012; Sassen, 2019). More in general, it is known that human dynamics are related to the diffusion of viral diseases (Eubank et al., 2004; Colizza et al., 2007; Perkins et al., 2014), to the behavioral responses in case of natural disasters (Bohorquez et al., 2009; Bagrow et al., 2011), to the optimization of traffic volumes (Batty, 2013; Mazzoli et al., 2019), to the economic growth, innovation and social integration (Bettencourt et al., 2007; Pan et al., 2013; Schläpfer et al., 2014), and to the severity of air pollution and the consumption of energy, water and other resources (Bettencourt et al., 2007; Bettencourt and West, 2010).

To monitor the progress toward the aforementioned societal challenges, it is fundamental to have an always up-to-date picture of cities. In the past, institutions had to rely almost exclusively on census data and official statistics. However, both these data sources have some intrinsic limitations including (i) the time gap between the data collection and the actual availability of the data, and (ii) the frequencies and costs of the data collection campaigns (Lazer et al., 2009). Luckily, we are in the middle of a digital sensing revolution with billions of data that are generated every second and that can be employed to have an almost real-time picture of cities' dynamics at low costs. Examples of such data include tracks from GPS devices embedded in smartphones, vehicles, or boats, records produced by the communication between phones and the cellular network, and geotagged posts from social media platforms (Gonzalez et al., 2008; Zheng et al., 2010; Moreira-Matias et al., 2013; Spinsanti et al., 2013; Blondel et al., 2015; Cui et al., 2018). Finally, other data sources like satellite images, street networks and points of interest can provide precious information to integrate with human dynamics data in order to capture socio-economic aspects (Deville et al., 2014; Jean et al., 2016; Tatem, 2017; Weber et al., 2018; Yeh et al., 2020; Lepri et al., 2022).

In this review paper, we showcase and discuss how alternative data sources have been employed by researchers to study the relationship between human dynamics and three SDGs: (i) crime diffusion and public safety, (ii) socio-economic inequalities and segregation, and (iii) public health and disease diffusion. Also, we have decided to focus on the studies that investigate such dynamics in urban agglomerations. Thus, excluding studies that, for example, investigate the relationship between international mobility and the global diffusion of diseases, or map the socio-economic inequalities across countries.

**Figure 1:**
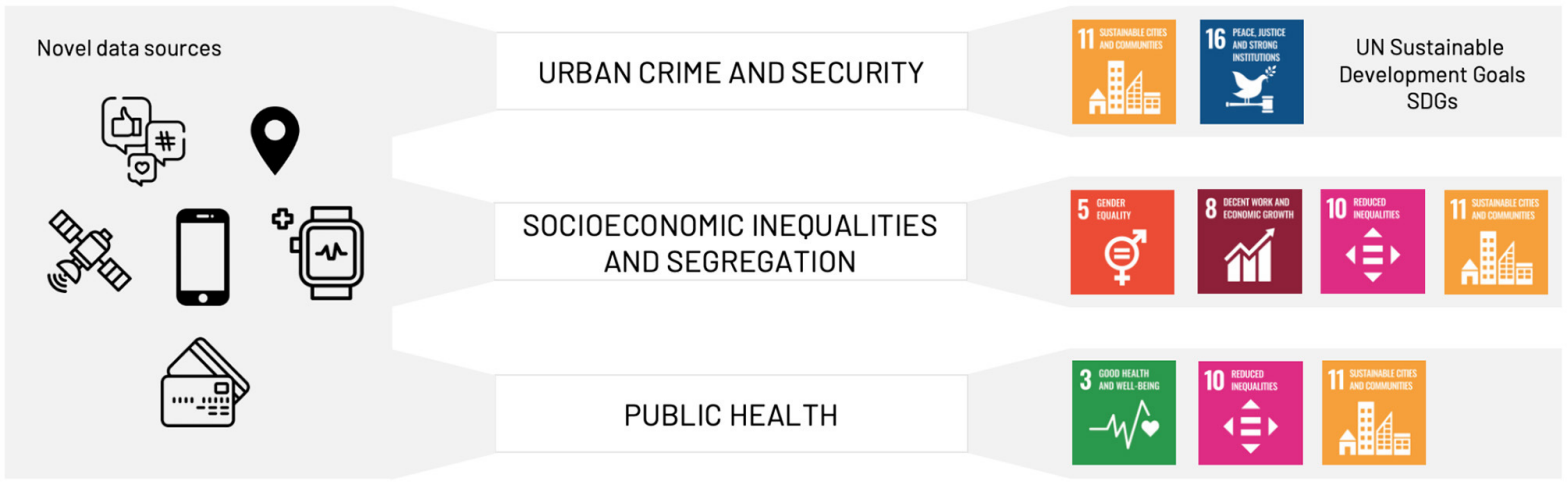
Visual summary of the topics covered in the paper. **(Middle)** The list of the addressed macro-topics and how they can be mapped to the United Nations' Sustainable Development Goals **(Right)**. **(Left)** An overview of the novel (alternative) data sources that enable the development of complex computational models that tackle problems in Urban Crime, Inequality and Public Health (icons: Flaticon.com; SDGs icons: UN).

Figure 1 provides a visual representation of the main topics covered in this review, highlighting how these topics are directly related to a set of SDGs. The main objective of this review paper is to examine how researchers make use of these novel digital data sources to develop new computational models and to derive insights that address important issues related to Urban Crime, Inequality and Public Health in urban environments.

The paper is structured as follows. We start in Section 2 by first describing the new sources of data used in these lines of research. Then, in Section 3, we discuss the implication of using computational methods for studying urban crime and public safety. After showing how researchers have used data sources like mobile phone data, social media, and others for a variety of aspects related to crime and security, we conclude the Section with a critical reflection. In Section 4 and Section 5, we follow a similar structure covering works related to socio-economic inequalities, segregation, and public health. Finally, in Section 6 we conclude the paper with a brief discussion.

## 2. Data sources

The digital age has revolutionized the world we live in, and simple actions such as clicking on a website, sending an email, paying with a credit card or making a phone call generate so-called digital traces. Digital traces track information about our daily behaviors and in the last decades this rich and vast amount of information created new research opportunities to better understand and study human behavior (Lazer et al., 2009, 2020). In this section we describe the most common type of data used in this line of research.

### 2.1. Call detail records

Telecommunication companies collect information regarding people's exchanges by means of Call Detail Records (CDRs), which contain real-world observations on how, when and with whom a person communicates (Blondel et al., 2015; Luca et al., 2021). A Call Detail Record is a tuple (u_*o*_, u_*i*_, *t, d, A*_*o*_, *A*_*i*_) which contains privacy-enhanced metadata about the caller u_*o*_, the callee u_*i*_, the timestamp *t* of when the call took place and its duration *d*. *A*_*o*_ and *A*_*i*_ represent respectively the outgoing and incoming Radio Base Stations (RBSs), namely the antennas that delivered the communication through the network. Mobile phone data may cover a large sample size on a national scale and aggregated mobility flows have been inferred by counting the number of users that move between RBSs or administrative units such as neighborhoods and municipalities (Calabrese et al., 2011). Since the position and the coverage area of each RBS are known, a user's telecommunication event represents a proxy of the user's geographic location. The precision of this location can vary from 100 m in urban areas to kilometers in rural areas. This approximation implies that the user's spatial and temporal resolution is given by where and when a user makes a call or sends an SMS leading to sparse and incomplete mobility trajectories. Nevertheless, CDRs have been proven valuable in studying and understanding human mobility (Gonzalez et al., 2008; Simini et al., 2012; Csáji et al., 2013; Blondel et al., 2015; Pappalardo et al., 2015).

A less common type of data used in the literature are the eXtended Detail Records (XDRs), which are generated by telecommunication companies when a user uploads or downloads data from the Internet using their phone's connection. A single event is a privacy-enhanced record (u, *t, A, k*) where *t* is the timestamp of the event, *A* is the RBS that managed the connection, and *k* the amount of uploaded/downloaded information. Given the higher frequency of mobile internet connections, XDRs reduce the problem of sparsity characterizing CDRs (Chen et al., 2019; Luca et al., 2022).

### 2.2. GPS location data

Location intelligence companies collect GPS location data of opt-in individuals from third-party mobile apps through a Software Development Kit (SDK) that captures user locations through GPS signals in Android and iOS devices. In general, a data point contains privacy-enhanced information like the *user identifier*, the *timestamp*, and geographic information such as *longitude* and *latitude*. In the last years, to help a prompt response to the COVID-19 pandemic, location intelligence companies such as Cuebiq,^3^ Unacast,^4^ and Safegraph^5^ made available several datasets for research purposes (Chang et al., 2021; Hunter et al., 2021; Lucchini et al., 2021; Aleta et al., 2022). The collected GPS location data generally provide more precise location information than CDRs. Unfortunately, location intelligence companies do not share details either on how the data is collected or from which mobile apps, potentially compromising their population representativeness.

On the same line, Big Tech companies such as Facebook (Data4Good^6^) and Google (Community Mobility Reports^7^) have provided GPS location data collected directly from their platforms. However, they have shared these data in an aggregated fashion both in time and space. Thus, the shared data have the advantage of covering all the countries the Big Tech companies operate in but are less precise than the GPS location data provided by location intelligence companies.

### 2.3. Social media data

Social media platforms such as Facebook, Instagram and Twitter facilitate the creation and sharing of content with posts that can contain text, videos, photos, etc. together with a timestamp and an (optional) geographic location. For example on Twitter, people can share geo-located tweets with either their precise geographical position (latitude and longitude) or the location suggested by the platform (e.g., restaurant, landmarks) where the user was located when the tweet was published. Platforms like Foursquare are location-based social networking websites where users share their locations by checking in at points of interest (POIs), such as restaurants, pubs, shops, museums. The users' location is thus available given that such venues have a geographical location (latitude and longitude). For most social media platforms, geotagged posts are downloadable through their Application Programming Interfaces (APIs). From spatial and temporal information present in social media posts is thus possible to infer users' mobility trajectories from their posts' history. Such data suffer from data sparsity problems with respect to datasets collected through mobile phones. Nevertheless, social media data have been proven valuable in modeling and dealing with different societal challenges such as urban crime, public health, unemployment (Wang et al., 2012; Broniatowski et al., 2013; Llorente et al., 2015).

### 2.4. Other data sources

#### 2.4.1. Credit card transactions

Credit cards are universal across the world, but they have received relatively little attention to date. Since people's spending has become increasingly digitized, it is possible to capture consumer behavior at an unprecedented scale. Each credit card transaction generally consists of privacy-enhanced information such as a *user identifier*, the *timestamp* of the transaction, and the *transaction type* represented by the Merchant Category Code (MCC). Recent research has begun to use transaction records to provide insights on financial wellbeing (Singh et al., 2015), individual traits (Gladstone et al., 2019; Tovanich et al., 2021), purchase behavior in urban populations (Dong et al., 2017; Di Clemente et al., 2018) and segregation (Dong et al., 2020).

#### 2.4.2. Satellite imagery

There exist several types of satellite imagery collected by governments and private companies and they can be mainly divided by their spatial, spectral, temporal, radiometric, and geometric resolutions (Campbell and Wynne, 2011). As an example, the Landsat Program represents the longest-running project for the acquisition of satellite imagery of Earth: they provide freely downloadable repeated (average return period of 16 days) imagery with a geometric resolution of 30 m for the entire planet. Satellite data has been proven useful for different tasks such as tracking urbanization (Tatem, 2017; Strano et al., 2021) and forecasting diseases (Dister et al., 1997; Rogers et al., 2002; Ford et al., 2009).

#### 2.4.3. Wearables

In the last few years, wearable sensors such as smartwatches have steadily grown in availability. These devices collect physiological and activity data such as heart rate, sleep, step count and calories burnt. This information can be exploited to track in almost real-time a person's health. As an example, recently smartwatches were used to investigate changes in physiological parameters in response to a COVID-19 infection or COVID-19 vaccines (Guan et al., 2022; Wiedermann et al., 2022).

#### 2.4.4. Census

Census data is collected by governments to monitor and gather information about the population of a country. The data is then used to have a reliable picture of the current population, including important information such as demographics and socio-economic conditions. Despite the vast amount of data produced in the digital age, census data remains widely used since it can be jointly used (at an aggregate level) with newer sources of data such as CDRs and provide valuable insights on the general population.

## 3. Urban crime and security

This section delves into the benefits of using alternative data sources to address urban crime and security challenges. The section first provides a brief overview of how the availability of novel data and computational models have altered the landscape of crime research (Section 3.1). Then, Sections 3.2 and 3.2.2 detail various computational methods researchers have employed. In Sections 3.2.3 and 3.2.4, we explore how mobile phone data and social media data have been employed in this line of research. Finally, we conclude the section with critical reflections and potential future directions.

### 3.1. The computational contamination in research on crime

Over the last two decades, scholars from various fields and disciplines have focused on crime and public safety by leveraging the potential of computational methods and novel big data sources. This wave of methodological innovation transcended the traditional boundaries of criminology as a discipline, fostering the interest of social scientists as well as computer scientists, statisticians, applied mathematicians and physicists. As a result, the study of crime has been invested by contamination of approaches, techniques, and viewpoints (Brantingham and Brantingham, 2004; Groff and Mazerolle, 2008; Bogomolov et al., 2015; D'Orsogna and Perc, 2015; Bouchard and Malm, 2016; Faust and Tita, 2019; De Nadai et al., 2020; Hayward and Maas, 2021; Campedelli, 2022b).

Interestingly, the link between computational methods and the study of crime is not as recent as many scholars portray. For instance, Campedelli (2022b) noted how, despite attempts to rebrand such a relationship in terms of novelty, the dialogue between Artificial Intelligence (AI) and research on crime has roots that date back to the 1980s. The relationship in fact emerged decades ago as the result of two processes: the use of AI-based approaches for predictive purposes (Icove, 1986) and the exploration of AI as a tool for aiding sociological theorizing (Brent, 1988; Anderson, 1989; Woolgar, 1989).

Hence, while it is limiting to describe the link between computational methods and the study of crime only by focusing on the recent past, it is nevertheless true that recent years have led to an acceleration in this dialogue, at least in terms of scientific productivity. The reasons behind this fact are four-fold. First, administrative data in digital format have become more and more ubiquitous and easy to access. Second, the democratization of programming languages made it easier for criminologists and crime researchers without a computer science background to explore the potential of algorithmic methods. Third, the availability of other digital sources such as social media data, GPS data, and mobile phone data enriched the information horizon available to study crime. Fourth, following a trend that was already in place, governments and institutions in many Western countries pushed for data-driven solutions to reduce crime, thus increasing funding opportunities in academia as well as business opportunities in the digital and technological sectors. All these factors together made it easier for scholars to gather, process, and analyze data related to crime and security issues, substantially increasing the number of publications and projects over the years (Campedelli, 2020).

Methodology-wise, crime has been investigated through a plethora of different techniques and frameworks. Besides traditional statistical approaches that target either correlational or causal outcomes, geospatial modeling, network science, agent-based modeling, and machine learning have been the four main areas on which scholars have focused their attention. Virtually every area of criminology and crime research has been—to some extent—explored by computational approaches: from white collar crime (Ribeiro et al., 2018; Luna-Pla and Nicolás-Carlock, 2020; Kertész and Wachs, 2021) to terrorism (Moon and Carley, 2007; Chuang et al., 2019; Campedelli et al., 2021), from illicit drugs (Mackey et al., 2018; Magliocca et al., 2019; Sarker et al., 2019) to organized crime (Nardin et al., 2016; Troitzsch, 2017; Calderoni et al., 2021), from gun violence (Mohler, 2014; Green et al., 2017; Loeffler and Flaxman, 2018) to cyber-crime (Shalaginov et al., 2017; Duxbury and Haynie, 2018, 2020), from recidivism (Tollenaar and van der Heijden, 2013; Duwe and Kim, 2017; Berk and Elzarka, 2020) to predictive policing (Caplan et al., 2011; Mohler et al., 2011; Perry, 2013). Particularly, the dialogue between computational methods and the study of recidivism and predictive policing not only focused on technical innovations to optimize forecasting and predictive models, but also provoked vivid debates regarding critical issues of algorithmic accountability, fairness, and transparency (Lum and Isaac, 2016; Dressel and Farid, 2018; Richardson et al., 2019; Akpinar et al., 2021; Purves, 2022). In fact, although the computational analysis of crime has remained chiefly confined to the academic sphere, in some cases—such as predictive policing and criminal justice risk assessment tools—algorithmic solutions have been deployed by courts and law enforcement agencies. In the US, where this transition from academia to the public and private sectors has been faster, data-driven tools to aid police agencies and courts have a long history (Berk, 2019). Yet, the rapid diffusion of novel tools, coupled with their secrecy, pushed scholars, activists and journalists to scrutinize the effects that these software have on high-stake settings, showing that these instruments often lead to disparate and unfair treatment against minorities, reinforcing discrimination and over-policing in policing and criminal justice. Two sides hence emerged: one populated by those defending the benefits and potential of computational approaches for predicting crime and recidivism (among other things), and those calling for either the elimination of such tools or their heavy regulation.

Within the kaleidoscope of areas in which the computational wave has spread, the study of urban crime has certainly fostered significant scholarly interest. Urban crime trivially embraces all those deviant and criminal behaviors occurring in urban settings and, therefore, can be seen as a higher-level category containing some of those previously mentioned, as the study of illicit drugs (when distributed or consumed in urban settings), the study of violent crime (when perpetrated in urban settings) or predictive policing itself, which by definition targets a certain urban area.

### 3.2. Computational methods, big data, and urban crime

#### 3.2.1. The advantages in studying urban crime today

There are some specific reasons behind the fact that urban crime has attracted so much scholarly attention. First and foremost, one of the most popular regularities in the empirical study of crime is the so-called “law of crime concentration” (Weisburd, 2015). Inspired by the theoretical tradition on crime and place (Shaw and McKay, 1942; Cohen and Felson, 1979; Eck and Weisburd, 1995; Brantingham and Brantingham, 2004), the “law of crime concentration” states that most crimes in a city are concentrated in specific small areas, such as blocks, streets or neighborhoods. In other words, crime clusters spatially (Johnson, 2010). The dawn of this empirical finding dates back to the early seminal cartographic works of Quetelet (1831) in the Nineteenth century. Over the decades, scores of studies emerged in the context of routine activity (Cohen and Felson, 1979) and crime pattern theories (Brantingham and Brantingham, 1984) have verified these findings not only in the US but in many other countries all around the world (De Melo et al., 2015; Ye et al., 2015; Mazeika and Kumar, 2017; Breetzke, 2018; Favarin, 2018; Umar et al., 2020). Second, not only does crime cluster spatially, it also clusters temporally. It is in fact well known that the probability that a crime occurs is not homogeneous across time windows (Aaltonen et al., 2018; Holbrook et al., 2021; Piatkowska and Lantz, 2021). In general, crime has its own higher-level seasonalities and these temporal dynamics also vary across crime types (Yan, 2004; Linning et al., 2017; Aaltonen et al., 2018). In many cases, these two layers intersect—especially in the case of urban crime—creating spatio-temporal regularities that allow for deeper analytical scrutiny. In general, spatial and temporal patterns create the conditions for deploying statistical and mathematical models taking advantage of the non-random data of criminal phenomena for forecasting and predictive purposes. A third reason behind the strong relationship between computational methods and urban crime is that both traditional and more novel data sources for studying crime both make spatial and temporal information available. In the US and other Western countries, for instance, data on reported crimes or calls for service are digitally recorded and easily accessible at the city level, combining information on the type of crime with information on the time and location of the offense. At the same time, data providers and tech companies sell or offer data on social media activity, mobile usage, public transportation, point of interest attendance, and GPS tracking. Overall, the availability of digital data beyond traditional administrative records has allowed scholars to expand the typical analytical frame in which crime—patterned but highly dynamic—is studied considering only fixed or static factors, variables, and conditions, such as the built environment or socio-economic characteristics. In light of this, the study of urban crime is aided by an arsenal of information that is rich—often way richer than the one available to the study of other crime contexts—and can be connected to other human phenomena that are known to be patterned, such as mobility flows. Fourth, the computational analysis of urban crime has straightforward practical consequences that transcend the pure research dimension. While the gap between empirical evidence and policy solutions may be wide for other areas of inquiry, the translation of empirical evidence to crime reduction strategies has always been much faster in the study of urban crime.

Scholars have taken advantage of these conditions and amassed a relevant number of studies with mainly two goals: disentangling crime correlates and forecasting or predicting crime trends and locations. The two goals are interrelated, as optimal forecasting can be achieved only through the selection of relevant correlates and, in turn, the study of correlates cannot be deemed independent from the need to optimize predictive performance.

#### 3.2.2. Mobility, urban crime, and ecological networks

Taxi flows and mobility patterns, as proxied for instance by the analysis of activity at POI locations, have been critical components of recent studies targeting urban crime. Some of the works emerging in this area have been framed using agent-based models (ABMs). ABM refers to generative models that allow research to simulate social and criminal phenomena by incorporating empirical or artificial data to investigate research questions that are impractical to be investigated in the real world (e.g., for ethical or monetary reasons). Although ABMs pose several major limitations to the reliability of findings when simulations are not appropriately designed and cannot be validated (Groff et al., 2019; Campedelli, 2022a), when models are carried out properly they offer a compelling set of benefits for criminologists and crime researchers, including theory testing, scenario exploration, and long-term forecasting.

Within this line of research, Ross et al. (2020) propose a simulation model for offender mobility in New York City (NYC) using open data to simulate urban structure, location-based information to proxy human activity and taxi flow data to proxy human mobility between different areas of the city. By comparing 35 different mobility patterns, the authors highlight the benefits of integrating taxi flow data with previous crime data and popular activity nodes to simulate offenders' mobility meaningfully. In another example, Ross et al. (2021) designed a model aimed at identifying drivers of relevant crime patterns through openly available static and dynamic geographical and temporal features, and proposed a data-driven decision-making process based on machine learning to allow artificial agents to decide whether to engage in crime based on their perception of the surrounding environment. Focusing again on NYC and targeting crime counts at the street level, the authors indicate the stability of high crime areas, in line with the criminological literature, and highlight the importance of the spatial environment in predicting crime hotspots. Agent-based modeling, however, is far from being the only methodological framework utilized in the study of urban crime through mobility data.

Wang et al. (2016), for instance, focused on Chicago to infer crime rates at the neighborhood level using POI and taxi flow data through more traditional statistical approaches like linear and negative binomial regression, indicating that including these information sources reduces prediction error by 17.6 percentage points. In a subsequent extension of the work, Wang et al. (2019) propose a graphically weighted regression approach for crime rate inference that aims at capturing the non-stationarity nature of crime across neighborhoods in the same urban context, i.e., Chicago, from 2010 to 2014. The assumption behind the analysis is that the same features may have different relationships to crime across different spatial contexts, thus involving a further layer of complexity in the dynamic nature of criminal phenomena. Chicago has been historically one of the US cities that attracted the highest scholarly attention in the study of deviance and crime (Sampson, 2012). In recent years, Chicago has also been the focus of several papers investigating crime from a computational perspective. Besides the articles mentioned above, others have explored the promises of sophisticated statistical techniques to shed light on the city's crime dynamics. Papachristos and Bastomski (2018), for instance, studied how criminal co-offending (measured via co-arrests data) generates pathways between neighborhoods in Chicago, creating a spatial network that facilitates the diffusion of crime in time and space. Their statistical analyses demonstrate that these “neighborhood networks” are stable over time, generated by various processes, including structural characteristics and social dynamics. Their work fits into a growing body of literature that assess the interdependency of neighborhoods within an urban context (Peterson and Krivo, 2009; Tita and Greenbaum, 2009; Graif et al., 2014), unfolding the connectivity of communities within cities, despite the belief that, given urban segregation and crime clustering, co-offending patterns should also be clustered. Relying on the interdependence of communities via spatial network representations, Graif et al. (2021) study the relationship between crime and commuting patterns across Chicago communities by concentrating on mobility patterns between job and home locations of the city residents. They mainly investigate whether exposure to workplaces characterized by higher disadvantage leads to an increase in local crime, suggesting that this relationship exists. In other words, Graif et al. (2021) shows that disadvantage in the extra-local network of communities where citizens work is associated with higher crime levels in the communities where the same citizens live. Sampson and Levy (2022) depart from a similar theoretical perspective to show that a neighborhood's wellbeing is statistically dependent upon the wellbeing of the communities that their residents visit or the communities from which visitors come. Remarkably, their analysis outlines that mobility-based socioeconomic disadvantage explains rates of violence and homicide in Chicago neighborhoods. The combination of low scores of residential socioeconomic conditions of residents, visited communities, and visitors constitutes what the authors label “triple disadvantage,” elaborating on how this concept is theoretically and technically valuable for explaining crime dynamics in Chicago (Levy et al., 2020).

#### 3.2.3. Urban crime and mobile phone data

Mobility and people dynamics within urban contexts have also been investigated by means of mobile phone data. One of the first notable examples is the work by Bogomolov et al. (2014) in which mobile network infrastructure data on London, UK, is combined with traditional demographic information and geo-localized open data to show that human behavioral data significantly improve the prediction of crime hotspots. London has been the focus of another early work by Traunmueller et al. (2014) in which anonymized mobile telecommunication data are used to investigate urban crime theories. From such telecommunication data, authors extract quantitative proxies for mapping the presence of people in a given area and find that the age diversity and the ratio of visitors in a given area are negatively related to crime, in line with theoretical concepts proposed by Jane Jacobs (Jacobs, 1961) such as the one of “natural surveillance” and by Felson and Clarke (Felson and Clarke, 1998), i.e., ratio of young people. Song et al. (2019) utilized geocoded tracks of mobile phones to analyze if the intensity of population mobility among pairs of communities in a large Chinese city can help shed light on offenders' decision-making processes. The study explicitly considers thefts, and its outcomes suggest that such a measure of mobility leads to a higher predictive performance of theft locations compared to the traditional analysis of crime generators. By leveraging mobile phone data and fine-grained spatio-temporal data on violent crime in Manchester, UK, Haleem et al. (2021) proposed the use of the “exposed population-at-risk” concept to shed light on public crime hotspots on Saturday nights. De Nadai et al. (2020) sought instead to examine the link of socioeconomic conditions, built environment and mobility patterns with violent and property crime across multiple cities. The authors identify the focus on single urban contexts as one of the main shortcomings of the existing literature. They hence focus on four contexts with very different social, cultural, and urban characteristics—i.e., Bogotá, Boston, Chicago, and Los Angeles—to provide higher external generalizability of their findings. Mobility flows are proxied through the use of mobile phone data in the form of CDRs. The work shows that combining information on people, crime, places, and human mobility produces better-performing models in terms of descriptive and predictive accuracy.

#### 3.2.4. The role of social media

In the thriving literature focusing on mobility and crime, recent studies have also sought to unfold the potential of social media to capture the dynamic dimension of human behavior in urban contexts, in line with the hypothesis emerging from other studies in the same area of research, namely that resident population does not explain the complexity of the ecology of crime. Among social media platforms, Twitter has certainly received greater attention from scholars interested in urban crime. Wang and Gerber (2015) proposed the use of Twitter data for solving the problem of “next-place prediction,” thus seeking to estimate people's individual trajectories. According to the authors, Twitter posts provide rich contextual information in the form of text that can be used to construct such individual trajectories even in cases when no direct reference to geospatial information is available. They hence present two models designed to extract geographic information from general texts allowing them to predict the type of venue a user will visit and the distance between the user and a given type of venue in the future. By leveraging this computational approach, they apply their methodology to test the correlation between next-place prediction and crime. Yang et al. (2018) included data from Twitter (and particularly data on the sentiment and topic of tweets) in their CrimeTelescope platform, a software intended to provide optimized crime hotspots prediction in New York. Besides Twitter data, CrimeTelescope also included information on urban infrastructure via POIs from Foursquare and historical crime data. The statistical outcomes of the study suggested that this multi-modal combination of data leads to better predictive performance (up to 5.2%) compared to traditional approaches only using data on past crimes. Malleson and Andresen (2015) highlighted that there is a relationship between the density of tweets in a given area and shifts in crime concentration. Similarly, Hipp et al. (2019) integrated geocoded Twitter data into models to capture the temporal ambient population in Southern California, arguing that social media data can be promising to test routine activity and crime pattern theories. Wo et al. (2022) examines the potential of four Twitter-derived measures to predict crime counts across more than 2,300 block groups in the city of Los Angeles. The aims of the study are specifically two. First, the authors seek to represent local human activity distinguishing between insider and outsider occupants of a neighborhood. Second, they analyze whether statistical relationships exist between property and violent crime and Twitter-derived measures of the ambient population in Los Angeles. Wo and co-authors conclude that Twitter is powerful in aiding research on ambient population and crime distributions at the spatial level. However, not all studies using Twitter data reached the same positive and promising conclusions. Tucker et al. (2021), in fact, critically tested whether geotagged Twitter data correlate with events of public violence and private conflict during weekday days, weekday nights, weekend days, and weekend nights in the city of Boston. The authors indicate that Twitter works as a proxy of human dynamics only for particular types of locations and activities, thus recommending caution in the use of tweets as comprehensive sources for mapping the ambient population within a city.

#### 3.2.5. Critical reflections

The study of crime has been impacted by the vast array of computational methodologies that have spread across social sciences in the last two decades, and urban crime in particular has benefited from this methodological contamination and the increasing availability of digital data sources coming from mobile phones, social media, transportation information, and other geolocalized trace data. This availability has opened many new possibilities to test criminological theories and improve predictive accuracy in terms of crime hotspots and crime patterns in space and time. Nonetheless, it should be noted that important caveats should be considered in critically evaluating the potential and relevance of novel data sources for the study of urban crime. Particularly, as noted by Browning et al. (2021), representativeness and generalizability of both mobility at the place- and person-level is a problem. Browning et al., for instance, argue that representativeness can be an issue when considering data that are collected based on voluntary choices of users and that this representativeness trivially poses a risk to the generalizability of findings across urban contexts (or even across areas within the same urban context). Hence, scholars must recognize this limitation and adopt strategies to mitigate it. Strategies may include methodological innovations in terms of weighting and result validation. Furthermore, the reliance on novel digital data may lead to an increase in the scholarly unbalance toward Western urban contexts. In fact, while accessibility to digital communication technologies is very high in the Western world, the scenario is very different for countries in other regions of the planet, reinforcing the abovementioned issue of representativity and generalizability when deploying these data sources outside the Western context. Finally, it is worth considering the societal implications of governmental decisions to incorporate mobile phones, GPS, POIs, and social media data into software designed for crime prediction, especially in non-democratic countries. In political contexts in which civil, political, and human rights are not sufficiently protected and guaranteed, the exploitation of multi-modal data sources may significantly increase the state of surveillance over citizens, causing detrimental effects on their liberties and wellbeing. Scholars should thus engage more in the ethical consequences of information systems engineered to collect as much data as possible to protect public safety and crime control allegedly.

## 4. Socioeconomic inequalities and segregation

This section explores how novel computational models can help in addressing socioeconomic inequalities and segregation-related issues. We organize the discussion as follows: Section 4.1 briefly summarizes how the increased availability of data and use of computational models benefit these research areas. Next, in Section 4.2, we describe novel computational methods developed to address socioeconomic inequalities and segregation with a focus on the role of GPS, mobile phones, and other data sources (Sections 4.2.1 and 4.2.2). Finally, we conclude the analysis of this line of research with critical reflections and future directions in Section 4.2.3.

### 4.1. The computational contamination in research on inequalities and segregation

Reducing inequalities is of crucial importance to guarantee a more sustainable and just future for our cities and societies. Indeed, socioeconomic inequalities and income segregation threaten access to health and negatively impact health population levels (Wilkinson and Pickett, 2006; Pickett and Wilkinson, 2015), prevent equal access to educational opportunities (Quillian, 2014; Logan and Burdick-Will, 2016), and hinder social and economic development (Neves et al., 2016). Moreover, they are intimately related to opportunities offered by neighborhoods (Sampson, 2012, 2019; Chetty et al., 2016; Manduca and Sampson, 2019; Hedefalk and Dribe, 2020) and human movements and interactions (Eagle et al., 2010; Chetty et al., 2016, 2022a,b; Wang et al., 2018; Dong et al., 2020). Before the advent of the digital era, socioeconomic inequalities were studied through surveys and census data. However, while census data provide a large-scale representativeness of the population, it lacks the ability to capture and provide an up-to-date picture of cities' dynamics and citizens' behaviors, routines, and habits (Lazer et al., 2009). As previously discussed, census data are collected every few years and are made publicly available several months after they were collected (Lazer et al., 2009). Instead, digital data provide alternative data sources that allow capturing different facets of human behavior: human interactions, human movements, and human encounters are just a few examples of human behavior which may play an essential role in investigating inequalities and segregation and that nowadays can be studied by means of mobile phone data (i.e., CDRs, GPS traces, etc.) and other digital traces (e.g., credit card transactions) (Lazer et al., 2009, 2020). In what follows, we discuss how several researchers, from economics and computational social science, have started using alternative data sources to study the daily behaviors and routines associated with socioeconomic inequalities and segregation in cities.

### 4.2. Computational methods, big data, and inequalities

As more people are moving to cities, governments have to deal with novel challenges like gentrification, unaffordability, segregation, and inequality (Glaeser et al., 2001; Florida, 2017). The place where a person lives can have substantial impacts on health (Wilkinson and Pickett, 2006), economic opportunities (Chetty et al., 2014, 2016), infrastructure and services accessibility (Glaeser et al., 2001; Reid et al., 2016; Florida, 2017), and many other aspects, both at a city and national scale (Chetty et al., 2014; Shelton et al., 2015). Thus, measuring inequalities and segregation with timely and accurate data is of paramount importance, and alternative data sources and ubiquitous technologies are starting to play a central role in deeply analyzing factors and behaviors associated to inequalities such as environmental inequalities (Brazil, 2022; Dass et al., 2022), social mixing and income segregation (Shelton et al., 2015; Moro et al., 2021; Fan et al., 2023), and community resilience (Hong et al., 2021).

#### 4.2.1. GPS and mobile phone data

Athey et al. (2020) developed a measure of experienced isolation (by race) to capture individuals' exposure to other (diverse) individuals using GPS data in the US. They found that the isolation individuals experience in their daily life is lower than the one measured by standard residential isolation metrics, especially in cities with higher levels of public transportation, density, education and income. Järv et al. (2015) moved beyond residential segregation to explore individuals' activity spaces, namely the locations visited by an individual because of their regular activities and routines. They exploited CDRs in the city of Tallin in Estonia to measure ethnic segregation (Estonian vs. Russian). They found that, for example, activity locations of Russian speakers tend to be more concentrated in regions with a prevalence of Russian-speaking communities. Xu et al. (2019) leveraged multiple urban datasets (e.g., CDRs records, housing prices and income data) to study citizens' segregation by their socio-economic status and its evolution in both physical and social (communication) spaces in Singapore. They found relatively higher levels of segregation in wealthier classes for both social and physical space. Hong et al. (2021) leveraged mobile phone data to measure the inequalities in community resilience to the Harvey hurricane in Texas. By measuring the mobility behavior of the individuals, the authors highlighted socio-economical and racial disparities in resilience capacity and evacuation patterns, suggesting the adoption of novel data-driven policies to prioritize equal allocations of resources to vulnerable neighborhoods. Another study (Dass et al., 2022) used mobile phone data, socio-demographic data, and infection rates information to measure accessibility to green spaces in Boston, at the beginning of the COVID-19 pandemic. The authors discovered inequalities, where communities with higher infections and higher prevalence of black residents experienced greater infection exposure per visit. Fan et al. (2023) employed mobile phone data of half a million people located in three different metropolitan areas in the US to study how people experienced social mixing in urban streets. The authors found that the density of people's street visits only explains the 26% of street-level diversity (e.g., social mixing), while the adjacent amenities, residential diversity, and income level explain the 44% of the designed diversity score. Also, Fan et al. (2023) shows that while streets densely visited tend to have more crime, diverse streets have fewer crimes. Moro et al. (2021) leveraged high-resolution mobility data of more than 4.5 million users in eleven big US cities to study income segregation. Previously, income segregation was studied using static residential patterns with high spatial resolutions. Thanks to the fine-grained mobility data, the authors found that the income segregation associated with places and individuals may significantly vary even for places that are close to each other. The authors proposed a model and showed that the experienced income segregation of individuals is associated with the exploration of new locations and places visited by visitors from different income groups. In general, Moro et al. (2021) highlights the importance of considering mobility patterns when we aim at measuring income segregation. Yabe et al. (2023) investigated how social interactions (e.g., encounters) changed during the COVID-19 pandemic with respect to income diversity. The authors relied on a dataset of millions of mobile phone users in multiple US cities for a period of three years before and during the pandemic. Overall, Yabe et al. (2023) found that the diversity of individual-level urban encounters decreased significantly despite the fact that in 2021, indices related to aggregated mobility recovered to pre-pandemic levels. The authors argued that the pandemic could have long-lasting implications on urban income diversity. Brazil (2022) utilized mobile phone data to study the mobility behavior of individuals to uncover environmental inequalities. The author found that people from minority groups and poorer neighborhoods tend to travel to areas with greater levels of air pollution with respect to white and richer neighborhoods.

#### 4.2.2. The role of other data sources

Using hundreds of millions of geotagged tweets, Wang et al. (2018) have measured neighborhood isolation for 50 American cities finding that residents of black and Hispanic neighborhoods, despite their socio-economic status, are less exposed to either non-poor or white neighborhoods than residents of white neighborhoods. Using the same dataset, Phillips et al. (2021) have computed the social integration of 50 American cities with indices that measure the extent to which residents in each neighborhood travel to other neighborhoods in a city. They have shown that cities with greater population densities and less racial segregation have higher levels of structural connectedness. Using Twitter data and geo-located credit card transactions Morales et al. (2019) investigated segregation in the physical and online space together with their relationship. They show that physical and online interactions in urban areas are segregated by income and that information does not flow homogeneously across social classes in either the physical or the virtual space. In a follow-up study, Dong et al. (2020) found that segregation in urban and online interactions seems stronger than the residential ones. Indeed, while residential neighborhoods sometimes might consist of a mix of different socioeconomic groups, purchase activities and online interactions seem to take place more often between neighborhoods whose economic conditions are similar. Additionally, Dong et al. (2020) have shown that segregation increases with differences in socioeconomic status but this effect is asymmetric for shopping behaviors. In fact, the number of movements from poorer to wealthier neighborhoods is larger than vice versa. Hilman et al. (2022) instead leveraged check-in datasets collected from location-based social media and census information to study segregation levels in 20 cities in the US. The authors found an upwards bias for which people of a certain socioeconomic class mostly visited places in the same class with rare visits to locations from higher classes. Furthermore, this bias increases with socioeconomic status and correlates with metrics for estimating racial residential segregation. In recent work, Shelton et al. (2015) showed that geotagged Twitter data can be used to capture the socio-spatial relations of territories dynamically. In particular, the authors analyzed the data for the city of Louisville in Kentucky, US, to show that analyzing the segregation of the city using static data is not sufficient to understand its dynamics.

Inequalities can also be related to the possibilities of accessing transportation. For example, by analyzing Boston's BlueBikers program mobility data, Fraser et al. (2022) showed that some neighborhoods use bike-sharing programs more than others. However, by considering the underlying socio-demographic characteristics, it emerged that there are significantly different adoption rates with respect to race and income level. Fraser et al. (2022) also pointed out how, by analyzing the mobility network over time (e.g., 2011–2021), Boston's program is gradually reaching a broader range of neighborhoods.

In a couple of recent works, Chetty et al. (2022a,b) have leveraged data from Facebook on 21 billion friendships. In particular, in a first study Chetty et al. (2022a) have measured three types of social capital by postal code in the US: (i) connectedness, namely friendship between people with different characteristics (i.e., high income vs low income), (ii) social cohesion, which is the extent to which networks of friends are clustered in cliques, and (iii) civic engagement, which measures as rates of volunteering or participation in civic organizations. Interestingly, the share of high-income friends among people with low income (called economic connectedness by the authors) is one of the more powerful predictors of upward economic (e.g., income) mobility. Moreover, Chetty et al. (2022a) have also found that differences in economic connectedness can explain upward income mobility, even when controlling for other strong neighborhood-level predictors such as poverty rates, racial segregation, and inequality. In a companion study and again using Facebook data, Chetty et al. (2022b) have shown that about half of the social disconnection across different socioeconomic groups is explained by differences in exposure to people with high socioeconomic status in places such as schools. Instead, the other half is explained by a friendship bias, namely a lower tendency of low-income people to establish friendships with high-income individuals. This ability to disentangle differences in exposures and friendship bias is of paramount relevance for building effective interventions and strategies to increase economic connectedness and thus decrease income segregation and inequalities.

#### 4.2.3. Critical reflections

New methodologies and novel insights on segregation and inequalities in urban environments have been developed thanks to novel data sources that enabled the study of different facets of human behavior and interaction at spatio-temporal scales that were previously unavailable. While these new approaches have brought substantial advantages for a timely study of human dynamics, it is still difficult to use these new methodologies and data sources to effectively measure the real impact of policies and interventions for reducing inequalities. In particular, this second aspect will require the development of a simulation framework able to generate different scenarios enabled by specific interventions. Moreover, given the proprietary nature of these novel data sources, it is often difficult to have access to longitudinal data that span multiple years and therefore it is problematic to track whether an intervention had an effective impact over time. An example of the potential of a study based on a longitudinal dataset is represented by Yabe et al. (2023), which using three years of data found that the COVID-19 pandemic still has long-lasting implications on urban income diversity in US cities. Furthermore, several studies are based on aggregated data and not on individual data for privacy reasons. This means that pieces of information such as poverty and deprivation are not directly available at the individual level and scholars have to develop proxies of such measures at an aggregated level (i.e., neighborhood) which may hinder the understanding of actual inequalities. As an example, Gündoğdu et al. (2019) had to move the two definitions of bridging and bonding social capital from an individual level to an aggregated level due to the unavailability of individual-level data.

## 5. Public health

In this section, we will examine how computational models can be useful in addressing public health-related issues. We will organize our discussion into several sections. First, we will briefly summarize how the availability of data and the use of computational models have impacted public health studies in Section 5.1. Then, we will analyze the benefits of using big data and new computational tools in Section 5.2.1. Next, we will explore how mobile phone data (Section 5.2.2), social media data (Section 5.2.3), and other data sources (Section 5.2.4) can be used to tackle these issues. Finally, we will wrap up this part of the research with a discussion of future directions and critical reflections in Section 5.2.5.

### 5.1. The computational contamination in research on public health

Public health is an inherently interdisciplinary field, whose key focus, preventing disease and promoting health, largely benefits from the contribution of different scholarly expertise, ranging from medicine to the social sciences, psychology and economics (Gavens et al., 2018). The past decades have seen an ever-increasing adoption of computational and digital methods in the research on public health, and many have been advocating for increasingly closer collaboration between computer scientists and public health scholars (Epstein, 2013).

In particular, major advances in the use of computational methods for public health have been witnessed in the area of infectious disease epidemiology, specifically to model and understand the patterns of disease dynamics and related causes. The use of mathematical models to study the spread of infectious diseases dates back to the seminal works of Ross (1916) and Kermack and McKendrick (1927) at the beginning of the 20th century, who first introduced the law of mass action in epidemiology. Over the years, epidemic models became increasingly complex, pursuing a higher level of realism by introducing additional interacting components, such as spatially defined structures (Rvachev and Longini, 1985; Sattenspiel and Dietz, 1995), age-stratified contact patterns (Fumanelli et al., 2012), human movements on long and short scales (Colizza et al., 2007; Balcan et al., 2009) and, more in general, human behavior (Funk et al., 2010; Perra et al., 2011). At the same time, the development of such models has been possible thanks to the increasing availability of computing power, thus allowing the *in-silico* recreation of populations with unprecedented levels of detail. If, in the origins of mathematical epidemiology, models were based on the assumption of a single, closed and well-mixed population (Grassly and Fraser, 2008), modern epidemic models are usually structured as ABMs, simulating the daily routines of up to hundreds of millions of individuals and their close contacts, in households, schools, and workplaces, requiring large-scale computational infrastructures (Ferguson et al., 2006; Ajelli et al., 2010; Merler and Ajelli, 2010).

The growth of computing power has been matched by even faster growth in data availability. With the diffusion of ubiquitous technologies and the rise of the Internet era, the field of epidemiology has been rapidly contaminated by digital approaches leading to a newly defined “digital epidemiology” (Salathe et al., 2012). Digital epidemiology, in the definition given by Salathé (2018), refers to epidemiology that uses data that was generated outside the public health system, data that were not collected with a specific public health-related purpose. The first study that brought to worldwide attention the potential use of a novel digital data source in epidemiology described Google Flu Trends, a system to monitor flu activity in more than 25 countries based on search query data (Ginsberg et al., 2009). The service was shut down in 2015, but historical data are still available. Also, the same study sparked a significant controversy on the accuracy of such emerging models and their potential biases (Lazer et al., 2014a,b). Soon thereafter, studies on digital epidemiology started growing exponentially, using a variety of digital sources to track disease prevalence and design public health interventions (Althouse et al., 2015; Bansal et al., 2016). Many studies followed the seminal example of Google Flu Trends by integrating different web data sources to forecast flu activity (Polgreen et al., 2008; Shaman and Karspeck, 2012; Shaman et al., 2013; Yuan et al., 2013; Lampos et al., 2015). As other data sources became rapidly available, their use has been explored in a wide range of epidemiological applications. Mobile phone data have been used to measure human movements and inform both spatially structured epidemic models (Tatem et al., 2009; Wesolowski et al., 2012, 2016), and surveillance systems (Barlacchi et al., 2017). Other studies have further advanced epidemic modeling and forecasting by combining additional data streams such as social media data (Lampos et al., 2010; Zhang et al., 2015, 2017), internet media reports (Freifeld et al., 2008), wearable sensors (Isella et al., 2011; Viboud and Santillana, 2020), and satellite imagery (Bharti et al., 2016; Castro et al., 2021). Finally, the opportunity provided by the Web to directly engage users in scientific research has opened the path to participatory surveillance systems, moving beyond the initial paradigm of passively collected data sources (Paolotti et al., 2014; Smolinski et al., 2015; Brownstein et al., 2017).

In this context, the COVID-19 pandemic has marked a turning point for digital epidemiology. While before 2020, digital epidemiology has been mainly studied as a proof-of-concept with a few real-time applications, since the early days of the COVID-19 outbreak in China, digital approaches have played a crucial role across the whole pandemic life cycle (Oliver et al., 2020), ranging from predictive modeling (Poletto et al., 2020) to the population-scale deployment of digital contact tracing apps (Colizza et al., 2021).

In the next sections, we discuss in more detail the role of the different alternative data sources and modeling techniques in computational epidemiology with a specific focus on applications in the urban context. First, we briefly highlight the general advantages of studying urban public health using digital approaches and big data. After that, we highlight the roles of mobile phone data, social media data, and other novel data streams.

### 5.2. Computational methods, big data, and urban public health

#### 5.2.1. The advantages of studying urban public health today

Modern epidemiology traces its roots to the foundational work of John Snow, who first identified the Broad Street pump as the source of the 1854 London cholera outbreak by mapping disease prevalence in Soho, and showing how cases occurred around this pump (Shiode et al., 2015). Since then, cartography and mapping spatial disease patterns have represented a fundamental tool for epidemiologists (Koch, 2011). In particular, the analysis of disease patterns in cities has attracted significant attention from scholars, as large metropolitan areas represent the main hubs of disease emergence and spreading (Ali and Keil, 2011; Connolly et al., 2021), a fact that has been well exemplified by the most recent global pandemics. Due to the high density of people and close proximity of living and working spaces in cities, infectious diseases can easily spread from person to person. Additionally, the crowded and often unsanitary living conditions in many cities can provide a conducive environment for the spread of infectious diseases. For example, inadequate access to clean water and sanitation facilities can lead to the proliferation of waterborne diseases such as cholera. Furthermore, the rapid pace of urbanization and population growth in many cities can put a strain on existing healthcare systems, making it more difficult to effectively detect and respond to outbreaks of infectious diseases (Neiderud, 2015).

In recent years scholars have investigated the role of urban features in the spread of infectious diseases, through a number of computational methods. On the one hand, computational epidemic models have been developed to capture the complexities of human behavior in the urban environment, from fine-scale human movements (Perkins et al., 2014) to contact networks (Eubank et al., 2004). On the other hand, many studies have explored the effect of city characteristics, such as urban population scaling laws (Bettencourt et al., 2007) on health outcomes (Rocha et al., 2015; Bilal et al., 2021) through a mix of theoretical and computational approaches (Schläpfer et al., 2014; Tizzoni et al., 2015). As digital trace data have become pervasive, providing researchers with a tool to investigate human behavior at a high spatial resolution, several studies have advanced our understanding of the role of urban structures in the spread of epidemics. By combining novel data sources with spatially resolved records of disease incidence, researchers have shown that variations in mobility patterns and the associated spatio-temporal fluctuations in population size can predict variations in the dynamics of seasonal flu epidemics (Dalziel et al., 2013, 2018; Zachreson et al., 2018). Similarly, the hierarchical structure of cities, and their different organization as single-center or multi-center systems, has been shown to predict inter-city variations in the spreading dynamics of respiratory infections (Rader et al., 2020; Brizuela et al., 2021; Aguilar et al., 2022).

Furthermore, the availability of high-resolution digital sources has also enabled the study of determinants of non-communicable diseases and chronic health conditions in urban areas. In particular, the analysis of digital trace data has allowed the characterization of neighborhoods based on novel behavioral indicators, thus providing new metrics to explain the observed residents' health outcomes (Sadilek and Kautz, 2013). Mobile phone data, social media data, and remote sensing have been used to model the pulse of urban life at a scale and granularity that would be hard to achieve with traditional methods. Overall, research in this area has demonstrated that novel digital sources represent an invaluable tool to monitor the health conditions of cities, understand their dynamics and inform public health policies. In the following sections, we provide an overview of some relevant contributions, based on different data sources, to address public health issues in large metropolitan areas.

#### 5.2.2. The role of mobile phone data

Location data generated by mobile phones have played a pivotal role in the modeling of human mobility and population settlements at international (Kraemer et al., 2020), national (Deville et al., 2014), and smaller spatial scales (Alessandretti, 2022). Since the early days of digital epidemiology, mobile phone data have represented an invaluable data source to connect empirical human mobility patterns and the spatial spread of infectious diseases. They have been used to calibrate epidemic models, understand disease spreading patterns, and evaluate intervention strategies against them (Wesolowski et al., 2016). Initial efforts to incorporate mobile phone derived mobility metrics into epidemic models have been mostly focused on large spatial scales, such as country-wide movements. This is the case, for instance, of seminal work by Tatem (2009), who leveraged mobile phone data collected in Zanzibar to estimate the relation between human mobility flows and parasite carrier movements and rates of malaria importation. The authors found that most of the people in Zanzibar traveled low-risk short distances but risk groups visiting higher-risk regions for extended periods could be identified. Similarly, Wesolowski et al. (2012) used mobile phone data and malaria prevalence information to estimate how people's movements were related to parasite importation between different regions. With their study, the authors were able to identify sources and sinks of imported infections and also to identify critical travel routes. Applications to city-scale epidemic scenarios have been generally more scarce, however, until the COVID-19 pandemic.

The COVID-19 pandemic has represented a defining moment for the use of mobile phone-derived data in epidemic modeling in cities. For the first time, high-resolution temporally resolved positioning data, collected from millions of users, became available to researchers. Such an unprecedented amount of information has fostered the development of a new generation of ABMs, with the ability to recreate synthetic populations of large urban areas with extraordinary realism, which was deemed impossible until a few years ago. While in 2011 Cooley et al. (2011) built their ABM of 7 million individuals living in New York City only based on the most recent census surveys, 10 years later, Aleta et al. (2020) could explicitly model the time-varying interactions of 100,000 people in the Boston Metropolitan Area, accounting for more than 5 million interactions in schools, workplaces, and households, derived from empirical co-location events. Their study showed that a response system based on enhanced testing and contact tracing could be an important tool to mitigate the spread of COVID-19, once social distancing measures were relaxed. In the following paper, Aleta et al. (2022) developed a similar model, integrating individual-level mobility data with socio-demographic information, to generate synthetic populations in New York City and Seattle, and simulate transmission events in more than 400,000 locations within the two cities. Such a detailed model allowed them to characterize the risk of COVID-19 transmission in different venues, identifying the most likely locations of super spreading events. In a similar effort, Chang et al. (2021) developed an epidemic ABM describing the mobility networks of ten of the largest US metropolitan areas. These networks mapped the hourly movements of 98 million people from census block groups, resulting in 5 billion dynamic edges. Simulations of COVID-19 spread on these large spatially-resolved networks demonstrated how higher infection rates among disadvantaged racial and socioeconomic groups were solely a result of differences in mobility in response to non-pharmaceutical interventions (NPIs). Studies of intra-city mobility during the pandemic were not limited to the US or Europe. As an example, Gozzi et al. (2021) investigated the dynamics of COVID-19 in the metro area of Santiago de Chile, using anonymized mobile phone data from 1.4 million users. By combining mobility traces and a compartmental epidemic model, they found that mobility responses to the lockdown were highly unequal in the city, with most deprived areas experiencing higher levels of mobility and, as a consequence, higher infection rates.

Other studies have used mobile phone data to investigate the socio-economic effects of NPIs in cities. Bonaccorsi et al. (2020) investigated the impact of the first lockdown measures in Italy, using a large-scale mobility dataset provided by Meta. They found that the impact on mobility was stronger in municipalities with higher fiscal capacity, while, at the same time, mobility reductions were larger in municipalities with higher income inequalities. Their results prompted fiscal interventions targeting the unequal effects of COVID-19 mitigation measures. On a similar note, Gauvin et al. (2021) used anonymized individual location data to study the mobility responses to COVID-19 in the neighborhoods of 3 major Italian cities. Their analysis uncovered the desertification of historic city centers, which persisted after the end of the first lockdown. Such a center-periphery gradient was mainly associated with differences in educational attainment. Similar results were found by Glodeanu et al. (2021) who evaluated socio-economic disparities of mobility responses in the neighborhoods of Madrid.

As mobility restrictions were removed and social life returned to normal, other studies focused on persistent changes in human behavior, that followed the pandemic response. For instance, Lucchini et al. (2021) used location data to analyze how people changed their mobility patterns and person-to-person contacts in response to NPIs in the US. Interestingly, they found a persistent reduction in close contacts and in the number of venues visited, even after the lifting of COVID-19 mandates. Using crowdsourced mobility data from 45 million devices, Li et al. (2022) found evidence of aggravated social segregation in the 12 largest US metropolitan areas, as a consequence of the COVID-19 mobility restrictions. Other studies, instead, have investigated the effects of the pandemic on lifestyles and individual habits. A notable example is a work by Hunter et al. (2021), who investigated the effects of the COVID-19 pandemic on walking habits in 10 major metropolitan areas of the US. The authors used individual-level mobility data to identify changes in the walking behavior of more than 1.6 million anonymized mobile phone users. Their findings highlighted a dramatic decline in walking habits during the first wave of the pandemic. Moreover, they found that once restrictions were lifted, walking levels recovered to pre-COVID-19 measures in high-income areas, whereas low-income areas were still well below pre-COVID-19 levels.

Finally, recent modeling advances have further developed the field of large-scale ABMs by combining high-resolution mobility data with detailed information on the economic role of individuals, as workers and consumers. Recent work by Pangallo et al. (2022) developed an ABM of the New York-Newark-Jersey City Metro Area that is representative of the real population across multiple socio-economic characteristics, including their employment status, the industry they work in, and their ability to work from home. Parameterizing the model with privacy-enhanced location data, the authors could explore the complex tradeoff between health and economy with an unprecedented level of realism.

#### 5.2.3. The role of social media

In digital epidemiology, social media data have always represented an important source of information to infer disease prevalence from health-related behaviors or symptoms reported by users (Brownstein et al., 2009; Aiello et al., 2020). Among social media, Twitter is the one that has attracted the most attention from scholars, thanks to the public availability, and machine readability, of basically all its content (Mejova et al., 2015b). The most typical use of Twitter data involves the automatic identification of relevant tweets, through either keyword search or natural-language processing, to identify posts whose content is related to some health condition (Paul and Dredze, 2011). For instance, tweets posted by users who report Influenza-Like Illness (ILI) symptoms. Collected tweets are then used as input to predictive models that aim at reproducing some known baseline, such as the ILI trends reported by official public health surveillance.

While initial efforts in this direction were mostly focused on measuring aggregated statistics of disease prevalence at the national level (Broniatowski et al., 2013; Gesualdo et al., 2013), the availability of geo-tagged social media data with GPS accuracy, in particular from Twitter, has allowed mapping users' health conditions at a very high spatial resolution, reaching the scale of a city (Sadilek et al., 2012). In a seminal paper, Nagar et al. (2014) used geo-referenced city-level Twitter data as a means of forecasting real-time ILI emergency department visits in New York City. They demonstrated that, at that spatial resolution, Twitter data could effectively capture the dynamics of flu in the boroughs of NYC. They also found that a model using the number of infection-related tweets outperformed one based on the number of web searches in predicting the number of ILI-related visits to emergency departments. Similarly, Lu et al. (2018) used Twitter data to model seasonal flu epidemics in the Boston Metropolitan Area.

Social media also represents a valuable data source to monitor non-communicable diseases and health habits in cities. A comprehensive study by Nguyen et al. (2016) developed a publicly available neighborhood-level dataset with indicators related to health behaviors and wellbeing in the USA. Interestingly, the authors found that greater happiness, and positivity toward physical activity and toward healthy foods, assessed via tweets, were associated with lower all-cause mortality and prevalence of chronic conditions such as obesity and diabetes and lower physical inactivity, and smoking. Similarly, Twitter data has been proven useful to map dietary habits in US cities, down to the level of census tract. A study by Gore et al. (2015) investigated how the obesity rate of an urban geographic area correlates with the contents of geo-tagged tweets in that area. In recent work by Sigalo et al. (2022), the authors analyzed about sixty-thousand geolocated food-related tweets collected across 25 cities, in the USA. They found associations between a census tract being classified as a food desert and an increase in the number of tweets in a census tract that mentioned unhealthy foods. Instagram, a video and photo sharing social network with more than 1 billion users worldwide, represents another relevant data source to investigate health habits, and in particular dietary choices, at scale. As an example, De Choudhury et al. (2016) showed that the textual content of Instagram posts predicts with high accuracy food deserts in the metropolitan areas of the US, while Mejova et al. (2015a) combined data from Instagram, Twitter, and Foursquare to correlate dietary choices and the prevalence of obesity across the USA. Another public health-relevant use of social media, which has been extensively explored by health departments in the US, is monitoring reports of foodborne illnesses. Two notable studies investigated the potential use of Twitter posts (Harris et al., 2014) and Yelp reviews (Harrison et al., 2014) to track food poisoning outbreaks in Chicago and New York City, respectively. Both studies demonstrated the high impact of using social media data to improve surveillance in collaboration with city public health authorities. Finally, Aiello et al. (2016) used a random sample of 17 million geo-referenced Flickr photos taken within Greater London between 2010 and 2015 to create a high-resolution map of the sound landscape of the city. They further leveraged such dataset to quantify the effects of noise on population health, correlating noise exposure levels with hypertension rates, at a very high spatial granularity (Gasco et al., 2020).

#### 5.2.4. The role of other data sources

Beyond mobile phone location data and social media, several studies have demonstrated the potential use of other digital sources for public health research. As already mentioned, multiple studies have leveraged search query data of services like Google Search (Ginsberg et al., 2009; Lampos et al., 2015), Baidu (Yuan et al., 2013), or Bing (Lampos et al., 2021), to monitor epidemics at scale. Internet search queries have not only been used to track the spread of infectious diseases but also to monitor other health conditions, for instance, mental health. As an example, Adler et al. (2019) combined official demographic statistics with data generated from Bing queries to gain insight into suicide rates per state in India as reported by the official census.

Another useful, but mostly untapped, data source to monitor disease incidence is Wikipedia pageview data. A landmark paper by McIver and Brownstein (2014) showed that the number of Wikipedia article views of specific health-related pages was a good predictor of ILI activity in the US. However, even though Wikipedia pageview data are geolocated, their availability with geo-encoded information is limited due to privacy reasons. Thus, city-scale studies are scarce. A notable example is a work by Tizzoni et al. (2020), who measured changes in awareness in the US during the 2016 Zika epidemic through geo-localized Wikipedia pageview data, at the level of US city. They examined the attention to Zika in 788 cities in the US with a population larger than 40,000 and found clear and distinct patterns of attention, varying with the exposure to the virus and the volume of media coverage.

Electronic records of retail market purchases represent a novel and interesting data stream, whose potential has been recently explored. Miliou et al. (2021) proposed to use retail market data to improve the forecasting of seasonal flu. In particular, the authors showed that by identifying some specific co-purchases of products, by specific customers, it is possible to model seasonal flu incidence in Italy, 4 weeks in advance, with improved accuracy with respect to an autoregressive baseline. Aiello et al. (2019) collected and analyzed a similar dataset, reaching an unprecedented level of spatial granularity. By analyzing 1.6 billion food item purchases and 1.1 billion medical prescriptions for the entire city of London over the course of one year, they showed that nutrient diversity and amount of calories are the two strongest predictors of the prevalence of three chronic health conditions: hypertension, high cholesterol, and diabetes.

#### 5.2.5. Critical reflections

The future of urban public health is undoubtedly going to be more and more digital. It is clear, however, that several challenges lie ahead, as has been evidenced by the adoption of computational and digital technologies during the COVID-19 pandemic. Thanks to increasing computing power and the availability of high-resolution behavioral data, computational models of epidemics are able to capture key determinants of transmission with impressive detail. However, they often lack a structural integration with socioeconomic dimensions that are known to affect epidemic outcomes. Recent studies have pointed out the need for equitable approaches in digital epidemiology, to address socioeconomic gaps in disease surveillance and modeling (Buckee et al., 2021; Tizzoni et al., 2022). Future work should aim at reducing health disparities during health emergencies through closer collaboration between epidemiologists and social scientists, psychologists, and economists.

Of course, the use of passively collected digital traces in public health comes with significant privacy and ethical concerns. In an urban context, measuring behaviors at a high spatial granularity represents a key advantage with respect to traditional data sources. However, reaching a high granularity may imply a higher risk of data re-identification, especially with small sample sizes, thus putting individual privacy at risk. In the future, it will be important to understand what privacy-preserving mechanisms can be most effective in minimizing such risks while preserving the potential of data analysis, even at a high spatial resolution.

Finally, the use of novel digital sources requires a careful understanding of their limitations and their scope. For instance, mobile phone-derived mobility metrics have been proven useful to understand the dynamics of COVID-19 in the early phase of the outbreak, however, the relationship between mobility indicators and epidemic outcomes is not straightforward (Kishore, 2021). While mobile phone data are clearly useful to measure changes in human behavior and link them with epidemic dynamics, such link often varies over time, and understanding this varying relationship poses significant challenges to scholars and policymakers who may want to use mobile phone data to evaluate the effectiveness of interventions or forecast future epidemic trajectories. Further work is needed to define methods that can systematically assess the quality of mobile phone-derived mobility metrics and make them comparable across different settings and data providers.

## 6. Conclusions

Cities are the beating heart of our modern societies. With more than half of the world's population living there, multiple emerging societal challenges require modern solutions. In particular, measuring the efficiency of deployed policies and progress toward specific SDGs, it is fundamental to have an always up-to-date picture of human dynamics in cities (e.g., how people move, how they interact with each other). In this context, it is clear that a pivotal role is played by the data collected from alternatives (ubiquitous) data sources like mobile phones, social media, GPS traces, satellite images, wearable devices and many others. In our review paper, we showcase how such alternative data are employed to monitor signs of progress toward some specific United Nations' Sustainable Development Goals. In particular, after a discussion about the different alternative data sources, we review how such information has been used to monitor urban crime and public safety. After that, we highlight the role of such data in reducing socioeconomic inequalities and segregation. Finally, we showed how they impacted research about public health. In all the sections, we start with a brief discussion about the advantages of using big data with respect to other techniques. Afterwards, we describe how different studies use such information. Finally, we conclude every section with some critical reflections about limitations and potential future directions.

## Author contributions

All authors listed have made a substantial, direct, and intellectual contribution to the work and approved it for publication.

## Footnotes

^1^ https://www.un.org/sustainabledevelopment/cities/

^2^ https://sdgs.un.org/goals

^3^ https://www.cuebiq.com/

^4^ https://www.unacast.com/

^5^ https://www.safegraph.com/

^6^ https://dataforgood.facebook.com/dfg/covid-19

^7^ https://www.google.com/covid19/mobility/
